# The impact of appendicular skeletal muscle index and trunk muscle index on stress urinary incontinence risk in female adults: a retrospective study

**DOI:** 10.3389/fnut.2024.1451400

**Published:** 2024-11-08

**Authors:** Junwei Wang, Cunming Zhang, Aiwei Zhang

**Affiliations:** ^1^Department of Urology, The Affiliated Wenling Hospital of Taizhou University (The First People’s Hospital of Wenling), Taizhou, China; ^2^Department of Ultrasound, The First People's Hospital of Wenling (Wenling Clinical College of Taizhou University), Taizhou, China

**Keywords:** stress urinary incontinence, appendicular skeletal muscle, appendicular skeletal muscle index, trunk muscle, trunk muscle index, sarcopenia, NHANES

## Abstract

**Objective:**

This study sought to examine the effect of the appendicular skeletal muscle index (ASMI) and trunk muscle index (TMI) on the likelihood of stress urinary incontinence (SUI) among female adults.

**Methods:**

This investigation utilized data from the National Health and Nutrition Examination Survey from 2001 to 2006 and 2011–2018. To evaluate the impact of ASMI and TMI on the likelihood of SUI, the study utilized restricted cubic splines (RCS) and weighted multivariable logistic regression models. Subgroup and interaction analyses were conducted to investigate how other covariates influenced their relationship.

**Results:**

In total, 11,168 female adults participated in the analysis. Multivariable logistic regression analysis revealed that high TMI was associated with a decreased likelihood of SUI (OR = 0.34; 95% CI: 0.16–0.75; *p* = 0.013). ASMI was not correlated with the likelihood of SUI. RCS analysis demonstrated a linear correlation between TMI and SUI risk, showing a decreasing trend in SUI risk as TMI increases (*p* for overall <0.001, *p* for nonlinearity = 0.73).

**Conclusion:**

Our study results showed that there was no association between ASMI and the risk of SUI, while a high TMI reduced the risk of SUI. This suggested that the ratio of muscle mass and BMI in different body regions has varying effects on SUI.

## 1 Introduction

Urinary incontinence (UI) is a frequently encountered condition affecting the lower urinary tract in adult women. Globally, more than 300 million women suffer from UI (1). Stress urinary incontinence (SUI) is among the most prevalent types of UI. It refers to the involuntary leakage of urine, often triggered by an increase in intra-abdominal pressure due to factors such as physical exertion, sneezing, or coughing (1, 2). Clinicians are not only focused on researching treatments for SUI but are also keen on studying its risk factors. Factors such as obesity, age, alcohol consumption, and smoking have been recognized as independent risk factors for SUI (3–5). By intervening in or avoiding these risk factors, some individuals with SUI may benefit from avoiding the economic burden and adverse effects associated with medication or surgical treatments. Therefore, the guideline regarded smoking cessation or weight loss as important conservative treatment methods for SUI (6).

The European Working Group on Sarcopenia in Older People (EWGSOP), the Foundation for the National Institutes of Health (FNIH), and the International Working Group on Sarcopenia (IWGS) had some differences in their diagnostic criteria for sarcopenia (7). In EWGSOP and EWGSOP2, the diagnosis of sarcopenia primarily referred to three factors: muscle mass, muscle strength, and gait speed, where appendicular skeletal muscle index (ASMI) was calculated by dividing appendicular skeletal muscle mass by height squared (8–10). In contrast, FNIH recommended calculating ASMI by dividing appendicular skeletal muscle mass by body mass index (BMI), a method that was considered to better adjust for differences in body size and to have a stronger association with muscle strength and physical function (7, 11). Sarcopenia is closely linked to diabetes and hypertension (10, 12–14), which may also increase the risk of UI (15, 16). Therefore, there may be a potential link between ASMI and SUI, which has not been studied before. Additionally, based on the ASMI calculation formula, we can see that ASMI reflects the balance between appendicular skeletal muscle mass, height, and weight, as appendicular skeletal muscle mass itself may be influenced by body size (17). Previous research has found a close association between limb skeletal muscle mass and abdominal muscle thickness (18, 19). The thickness and function of abdominal muscles may also potentially influence the occurrence of SUI (20–22). Therefore, whether ASMI is associated with the risk of SUI needs further investigation and exploration.

However, it is well known that SUI is more likely to be associated with pelvic muscle function (23). Therefore, we hypothesize whether the ratio of pelvic muscle mass to BMI is related to the risk of SUI. However, in the National Health and Nutrition Examination Survey (NHANES), there is no separate data for pelvic muscle mass as it is included in trunk muscle mass. Therefore, in this study, we used the ratio of trunk muscle mass to BMI, named the trunk muscle index (TMI), to investigate its relationship with the risk of SUI. Additionally, the activity of trunk muscles may influence the occurrence of SUI due to posture correction or balance abilities (24). Therefore, trunk stabilization exercises for pelvic floor muscles may be beneficial in improving symptoms of postpartum urinary incontinence (25). Thus, the connection between trunk muscles and SUI is also of great interest.

The relationship between muscle mass or strength and urinary incontinence has also been a focus of attention. One study found that a high total muscle-to-fat ratio may reduce the likelihood of urinary incontinence (26). When muscle mass is adjusted for body weight or BMI, there is a significant correlation between urinary incontinence and low muscle mass (27). However, these studies often lack research linking specific muscle quality indices to SUI, as the physiological functions of muscle quality or function may differ across various regions. Similarly, the occurrence of SUI may also be influenced by the muscle quality in different regions.

This study collected data on adult women from the 2001–2006 and 2011–2018 NHANES, aiming to assess the relationship between ASMI, TMMI, and SUI risk, thereby providing new perspectives for the treatment and management of SUI patients.

## 2 Materials and methods

### 2.1 Date and participants

The data used in this study came from NHANES,^1^ and provided survey methods and analysis guidelines.^2^ NHANES is a significant nationwide survey project conducted by the National Center for Health Statistics (NCHS), under the Centers for Disease Control and Prevention (CDC) in the USA. It employs a stratified, multistage sampling technique to collect medical and nutritional data from the Americans, ensuring that the sample is nationally representative. Additionally, all participants provided signed consent forms, and the study received approval from the Research Ethics Review Board of the National Center for Health Statistics. This study obtained survey data from NHANES for the years 2001–2006 and 2011–2018.

**Figure 1:**
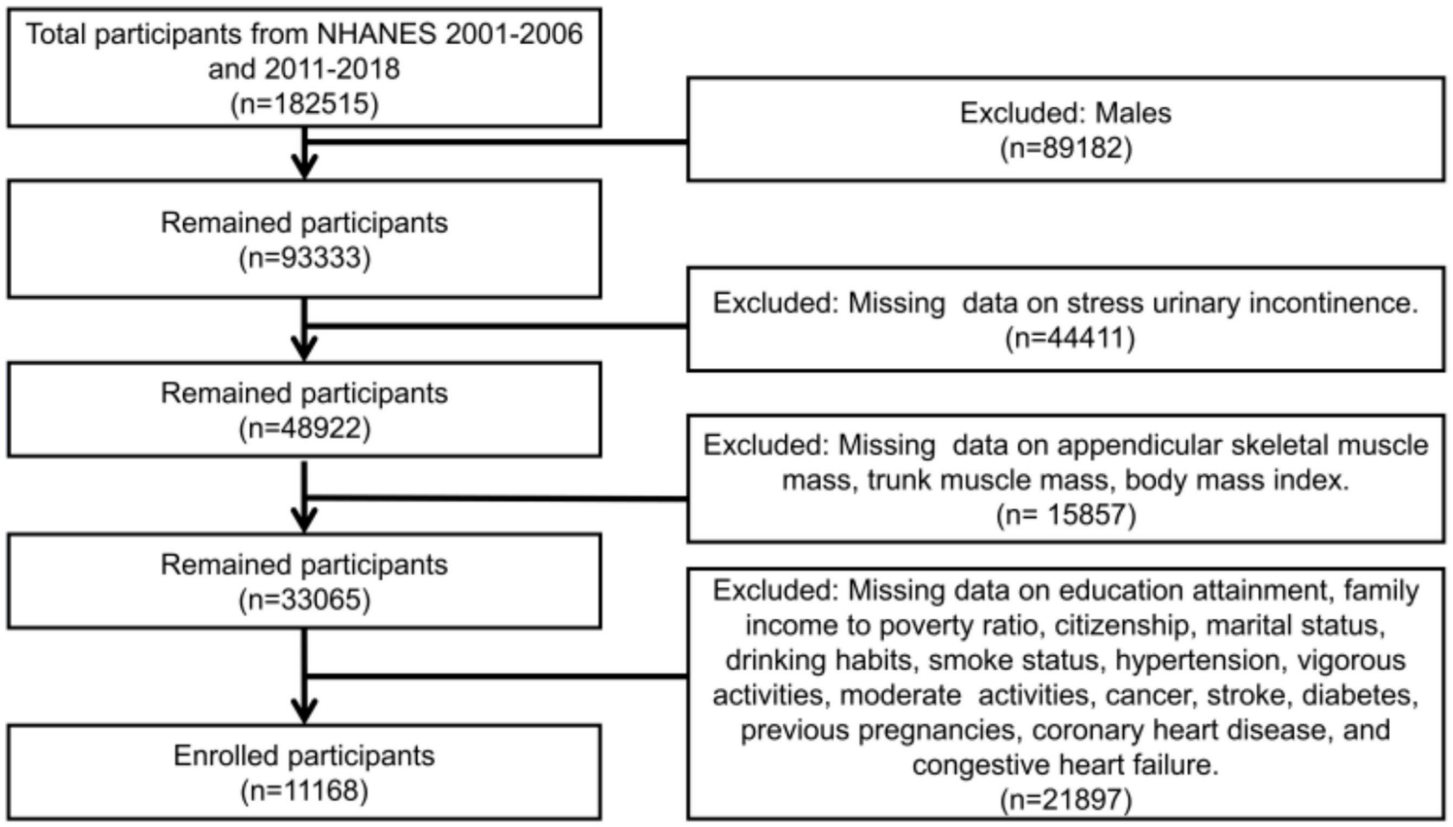
Flowchart for participant selection.

The criteria for participation in this study were adult female participants aged 20 and above with complete data. The exclusion criteria included individuals with missing data on SUI, appendicular skeletal muscle mass, trunk muscle mass, BMI, and other covariates. Additionally, appendicular skeletal muscle mass and trunk muscle mass were obtained through Dual-energy X-ray Absorptiometry (DXA) scans, which have their own exclusion criteria. These criteria encompass pregnancy, as indicated by a positive urine pregnancy test and/or self-reported pregnancy at the time of the DXA scan. These criteria also include self-reported usage of radiographic contrast agents (such as barium) within the previous 7 days, as well as self-reported body weight exceeding 450 pounds or height exceeding 6 feet 5 inches, which are beyond the limits of the DXA table. Based on these criteria for inclusion and exclusion, a total of 11,168 participants were included in this study (Figure 1).

### 2.2 Exposure and outcome variables

ASMI and TMI were the exposure variables in this study. The calculation formula for ASMI was appendicular skeletal muscle mass (kg) divided by BMI (kg/m^2^); whereas the calculation formula for TMI was trunk muscle mass (kg) divided by BMI (kg/m^2^). The skeletal and soft tissue measurements of the arms, legs, pelvis, left and right ribs, thoracic spine, and lumbar spine were obtained through DXA scans. Appendicular skeletal muscle mass comprised arm and leg muscle mass. Trunk muscle mass included pelvis, ribs, thoracic spine, and lumbar spine muscles. It should be noted that these values did not include bone mineral content. Although the main focus of this study was on the muscle mass of the pelvis, the NHANES data did not provide separate muscle mass data for the pelvis, and it was instead included in the trunk muscle mass. BMI values were directly obtained from the Body Measures section in the Examination Data of NHANES. SUI was the outcome variable in this study, defined based on the following questionnaire: “During the past 12 months, have you leaked or lost control of even a small amount of urine during activities such as coughing, lifting, or exercise?” Individuals who responded ‘yes’ were classified as having SUI, while those who responded ‘no’ were classified as not having SUI.

### 2.3 Covariates

Drawing from prior research, this study extracted various covariates from the NHANES database. Age was classified into three categories: 20–39, 40–59, and 60 years and above. Educational attainment was divided into three levels: below high school level, high school level, and above high school level. Race was divided into the following groups: Mexican American, Non-Hispanic Black, Non-Hispanic White, Other Hispanic, and Other Race. Drinking habits were sorted into three groups: up to 5 drinks per day, 6–10 drinks per day, and more than 10 drinks per day. The family income to poverty ratio (PIR) was categorized as less than 1.3, between 1.3 and 3.4, and 3.5 or higher. Smoking status was classified into three categories: never smoked (fewer than 100 cigarettes in their lifetime and not currently smoking), former smokers (at least 100 cigarettes in their lifetime but had quit), and current smokers (at least 100 cigarettes in their lifetime and still smoking). Marital status is divided into the following groups: divorced, living with partner, married, never married, separated, and widowed. Diabetes or hypertension was considered present if diagnosed by a healthcare professional or if relevant medications were being used. Vigorous activity was defined as engaging in at least 10 min of intense activity that resulted in significant sweating or a substantial increase in breathing or heart rate. Moderate activity was defined as engaging in at least 10 min of moderate activity that resulted in only slight sweating or a slight to moderate increase in breathing or heart rate. Previous pregnancies were divided into “Yes” and “No” groups. If the participants engaged in vigorous or moderate activity, they answered yes; otherwise, they answered no. The assessment of cancer, coronary heart disease, congestive heart failure, and hysterectomy, relied on self-report in the Questionnaire, with the question “Has a doctor or other health professional ever informed you of having [respective condition]?”

### 2.4 Statistical analysis

In this study, all participants were divided into two groups: those with SUI and those without SUI. Given the large sample size in this study and based on the Central Limit Theorem (28), we utilized the Student’s t-tests to compare continuous variables. Continuous variables were represented by mean ± standard deviation (SD), whereas categorical variables were expressed as proportions (%) and frequencies (n). Differences in demographic data between the two groups were analyzed using weighted Student’s t-tests and chi-square tests. TMI was analyzed not only as a continuous variable but also categorized into weighted quartiles. Multiple logistic regression models were used to estimate the relationship between ASMI, TMI, and SUI, expressed as odds ratios (OR) and corresponding 95% confidence intervals (CI). Model 1 did not include any covariates, Model 2 incorporated covariates including age, race, PIR, marital status, citizenship, and education attainment. Model 3, building on Model 2, added smoking status, drinking habits, hysterectomy, cancer, stroke, vigorous activities, moderate activities, previous pregnancies, coronary heart disease, congestive heart failure, diabetes, and hypertension. A restricted cubic spline curve was used to present the relationship between TMI and SUI. Utilizing Model 3, which accounted for all covariates, the connection between TMI and the risk of SUI was illustrated using restricted cubic splines (RCS). The main advantage of RCS is its ability to flexibly capture the nonlinear relationships between variables, thereby providing more accurate results. This method improves the interpretability of the data and performs well in avoiding overfitting. Subgroup analyses were conducted based on age, educational attainment, PIR, smoking status, vigorous activities, and hysterectomy to assess whether these factors influenced the relationship between TMI and the risk of SUI. All analyses were performed using R software (version 4.2.3). Differences were considered statistically significant when the *p* value was less than 0.05.

## 3 Results

### 3.1 Demographic characteristics

As shown in the flowchart (Figure 1), a total of 11,168 female adult participants aged 20 and above were enrolled in the final analysis of this study, including 6,239 individuals without SUI and 4,929 individuals with SUI. Table 1 presented the baseline characteristics of the two groups. We found that in the SUI group, the proportions of individuals who were over 40 years old, Non-Hispanic White, did not engage in vigorous activities, had hysterectomy, had hypertension, and were married were higher. Additionally, the SUI group had higher levels of appendicular skeletal muscle, trunk muscle, and BMI, but lower levels of ASMI and TMI.

**Table 1:** Participants’ clinical characteristics obtained from the National Health and Nutrition.

Variables	Without SUI	With SUI	*p* value
	(*n* = 6,239)	(*n* = 4,929)	
Appendicular skeletal muscle (kg)	17.67 ± 0.10	18.08 ± 0.13	0.01
Trunk muscle (kg)	21.45 ± 0.11	22.34 ± 0.14	<0.0001
BMI (kg/m^2^)	27.55 ± 0.19	29.30 ± 0.23	<0.0001
Appendicular skeletal muscle index [kg/(kg/m^2^)]	0.65 ± 0.00	0.63 ± 0.00	<0.0001
Trunk muscle index [kg/(kg/m^2^)]	0.80 ± 0.00	0.78 ± 0.00	<0.001
Age, *n* (%)			<0.0001
20–39 years	2095 (34.8)	826 (18.3)	
40–59 years	2,589 (48.8)	2,463 (58.5)	
≥60 years	1,555 (16.4)	1,640 (23.2)	
Race, *n* (%)			<0.0001
Mexican American	805 (5.0)	778 (5.1)	
Non-Hispanic Black	1,438 (12.0)	633 (6.2)	
Non-Hispanic White	3,231 (73.9)	3,033 (80.6)	
Other Hispanic	317 (4.5)	212 (3.3)	
Other Race	448 (4.7)	273 (4.8)	
Educational attainment, *n* (%)			0.06
Below high school level	1,038 (9.8)	865 (11.6)	
College level and above	3,776 (67.3)	2,773 (62.2)	
High school level	1,425 (22.9)	1,291 (26.1)	
PIR, *n* (%)			0.61
<1.3	1,348 (15.3)	1,108 (16.0)	
1.3–3.4	2,354 (34.1)	1734 (32.6)	
≥3.5	2,537 (50.6)	2087 (51.5)	
Smoke status, *n* (%)			0.34
Former	1,396 (24.0)	1,327 (25.4)	
Never	3,568 (53.7)	2,485 (50.3)	
Current	1,275 (22.4)	1,117 (24.3)	
Drinking habits, *n* (%)			0.44
≤ 5 drinks/day	6,059 (97.5)	4,776 (96.8)	
6–10 drinks/day	158 (2.2)	132 (2.8)	
≥11 drinks/day	22 (0.3)	21 (0.4)	
Vigorous activities, *n* (%)			< 0.001
No	4,410 (67.1)	3,824 (73.9)	
Yes	1829 (32.9)	1,105 (26.1)	
Moderate activities, *n* (%)			0.82
No	2,883 (40.3)	2,317 (40.8)	
Yes	3,356 (59.7)	2,612 (59.2)	
Hysterectomy, *n* (%)			< 0.0001
No	4,585 (74.6)	3,140 (64.8)	
Yes	1,654 (25.4)	1789 (35.2)	
Cancer, *n* (%)			0.12
No	5,686 (90.0)	4,265 (87.3)	
Yes	553 (10.0)	664 (12.7)	
Stroke, *n* (%)			0.05
No	6,123 (98.5)	4,773 (97.0)	
Yes	116 (1.5)	156 (3.0)	
Diabetes, *n* (%)			0.29
No	5,776 (94.3)	4,437 (93.3)	
Yes	463 (5.7)	492 (6.7)	
Hypertension, *n* (%)			< 0.0001
No	4,299 (73.0)	3,000 (65.1)	
Yes	1,940 (27.0)	1,929 (34.9)	
Coronary heart disease, *n* (%)			0.47
No	6,124 (98.4)	4,805 (98.0)	
Yes	115 (1.6)	124 (2.0)	
Congestive heart failure, *n* (%)			0.24
No	6,175 (99.2)	4,820 (98.7)	
Yes	64 (0.8)	109 (1.3)	
Marital status, *n* (%)			< 0.0001
Divorced	791 (13.0)	729 (13.9)	
Living with partner	462 (7.6)	249 (4.4)	
Married	2,885 (52.3)	2,768 (63.3)	
Never married	1,220 (17.4)	399 (6.9)	
Separated	237 (2.8)	169 (2.5)	
Widowed	644 (6.9)	615 (9.0)	
Citizenship, *n* (%)			0.06
No	498 (4.9)	287 (3.3)	
Yes	5,741 (95.1)	4,642 (96.7)	
Previous pregnancies, *n* (%)			< 0.0001
No	1,265 (22.8)	434 (9.3)	
Yes	4,974 (77.2)	4,495 (90.7)	
Trunk muscle index			0.003
Quantile1 (0.46,0.69)	1,429 (18.4)	1,354 (22.0)	
Quantile2 (0.69,0.75)	1,498 (22.0)	1,292 (23.1)	
Quantile3 (0.76,0.83)	1,561 (25.6)	1,248 (28.2)	
Quantile4 (0.84,1.25)	1751 (34.0)	1,035 (26.8)	

Data are expressed as means ± SD or number (%). *χ*^2^ test for categorical variables. T-test for continuous variables. Abbreviations: BMI, body mass index; PIR, family income to poverty ratio; SUI, stress urinary incontinence.

Participants who were excluded from the study due to missing data differed in some characteristics from those who were successfully included in the study. Compared to the excluded group, the included group had a higher proportion of individuals who were aged 40–59 and 60 years and older, a higher proportion of non-Hispanic whites, a lower proportion of individuals who had education below high school, a lower proportion of those who had never smoked, a higher proportion of individuals who drank less than 5 drinks per day, and a higher proportion of individuals who had a history of pregnancy (Table 2).

**Table 2:** Comparison of characteristics between participants excluded due to missing data and those included in the study.

Variables	Included group	Excluded group	*p* value
	(*n* = 11,168)	(*n* = 21,897)	
Appendicular skeletal muscle (kg)	17.86 ± 0.09	17.95 ± 0.09	0.38
Trunk muscle (kg)	21.86 ± 0.10	21.73 ± 0.09	0.28
BMI (kg/m^2^)	28.34 ± 0.16	28.48 ± 0.18	0.51
Appendicular skeletal muscle index [kg/(kg/m^2^)]	0.64 ± 0.00	0.64 ± 0.00	0.65
Trunk muscle index [kg/(kg/m^2^)]	0.79 ± 0.00	0.78 ± 0.00	0.17
Age, *n* (%)			< 0.0001
20–39 years	2,921 (27.3)	8,645 (45.3)	
40–59 years	5,052 (53.2)	7,687 (38.9)	
≥60 years	3,195 (19.5)	5,565 (15.8)	
Race, *n* (%)			< 0.0001
Mexican American	1,583 (5.0)	4,711 (8.0)	
Non-Hispanic Black	2,071 (9.3)	4,916 (12.7)	
Non-Hispanic White	6,264 (77.0)	10,042 (67.6)	
Other Hispanic	529 (4.0)	986 (5.4)	
Other Race	721 (4.7)	1,242 (6.3)	
Educational attainment, *n* (%)			< 0.0001
Below high school level	1,903 (10.6)	6,450 (18.9)	
College level and above	6,549 (65.0)	10,434 (57.2)	
High school level	2,716 (24.4)	4,977 (23.9)	
Missing	0 (0.0)	36 (0.1)	
PIR, *n* (%)			< 0.0001
<1.3	2,456 (15.6)	6,393 (22.4)	
1.3–3.4	4,088 (33.4)	7,904 (35.5)	
≥3.5	4,624 (51.0)	5,625 (34.3)	
Missing	0 (0.0)	1,975 (7.8)	
Smoke status, *n* (%)			< 0.0001
Former	2,723 (24.6)	3,814 (18.1)	
Never	6,053 (52.1)	14,090 (60.9)	
Current	2,392 (23.2)	3,966 (20.9)	
Missing	0 (0.0)	27 (0.1)	
Drinking habits, *n* (%)			< 0.0001
≤ 5 drinks/day	10,835 (97.2)	8,305 (46.8)	
6–10 drinks/day	290 (2.4)	392 (2.3)	
≥ 11 drinks/day	43 (0.4)	48 (0.3)	
Missing	0 (0.0)	13,152 (50.6)	
Vigorous activities, *n* (%)			0.02
No	8,234 (70.2)	16,029 (67.0)	
Yes	2,934 (29.8)	5,853 (33.0)	
Missing	0 (0.0)	15 (0.0)	
Moderate activities, *n* (%)			0.01
No	5,200 (40.5)	11,329 (44.3)	
Yes	5,968 (59.5)	10,548 (55.7)	
Missing	0 (0.0)	20 (0.0)	
Hysterectomy, n(%)			< 0.0001
No	7,725 (70.1)	6,320 (23.0)	
Yes	3,443 (29.9)	3,733 (13.6)	
Missing	0 (0.0)	11,844 (63.4)	
Cancer, *n* (%)			< 0.0001
No	9,951 (88.7)	20,201 (92.1)	
Yes	1,217 (11.3)	1,624 (7.5)	
Missing	0 (0.0)	72 (0.3)	
Stroke, *n* (%)			0.15
No	10,896 (97.8)	21,103 (97.2)	
Yes	272 (2.2)	758 (2.7)	
Missing	0 (0.0)	36 (0.1)	
Diabetes, *n* (%)			0.004
No	10,213 (93.8)	19,375 (91.8)	
Yes	955 (6.2)	2,522 (8.2)	
Hypertension, *n* (%)			< 0.001
No	7,299 (69.4)	14,839 (72.9)	
Yes	3,869 (30.6)	6,925 (26.6)	
Missing	0 (0.0)	133 (0.6)	
Coronary heart disease, *n* (%)			0.01
No	10,929 (98.3)	21,129 (97.4)	
Yes	239 (1.7)	615 (2.1)	
Missing	0 (0.0)	153 (0.5)	
Congestive heart failure, *n* (%)			< 0.001
No	10,995 (99.0)	21,131 (97.6)	
Yes	173 (1.0)	675 (2.1)	
Missing	0 (0.0)	91 (0.3)	
Marital status, *n* (%)			< 0.0001
Divorced	1,520 (13.4)	2,340 (10.6)	
Living with partner	711 (6.2)	1,407 (7.2)	
Married	5,653 (57.3)	10,759 (53.4)	
Never married	1,619 (12.6)	3,834 (18.2)	
Separated	406 (2.7)	879 (3.1)	
Widowed	1,259 (7.8)	2,662 (7.4)	
Missing	0 (0.0)	16 (0.1)	
Citizenship, *n* (%)			< 0.0001
No	785 (4.2)	3,154 (9.2)	
Yes	10,383 (95.8)	18,713 (90.8)	
Missing	0 (0.0)	30 (0.1)	
Previous pregnancies, *n* (%)			< 0.0001
No	1,699 (16.7)	3,638 (20.9)	
Yes	9,469 (83.3)	17,773 (77.8)	
Missing	0 (0.0)	486 (1.3)	

Data are expressed as means ± SD or number (%). *χ*2 test for categorical variables. T-test for continuous variables. Abbreviations: BMI, body mass in dex; PIR, family income to poverty ratio; SUI, stress urinary incontinence.

### 3.2 Association between ASMI, TMI, and SUI

To verify the relationship between ASMI, TMI, and SUI, we conducted logistic regression analysis using three models (Table 3). In Model 1, which did not adjust for any covariates, the risk of SUI decreased by 92% for each unit increase in ASMI (OR = 0.08; 95% CI: 0.03–0.20; *p* < 0.001). A comparable correlation was noted in Model 2, which accounted for covariates including age, race, PIR, marital status, citizenship, and educational attainment. In this model, the risk of SUI decreased by 69% for each unit increase in ASMI (OR = 0.31; 95% CI: 0.11–0.93; *p* = 0.04). However, in Model 3, which included all covariates, the relationship between ASMI and the risk of SUI disappeared (*p* = 0.06).

**Table 3:** Association between appendicular skeletal muscle index, trunk muscle index and stress urinary incontinence.

Exposure	Model 1		Model 2		Model 3	
	OR, 95%CI	*p*	OR, 95%CI	*p*	OR, 95%CI	*p*
Appendicular skeletal muscle index [kg/(kg/m^2^)]						
Continuous variable	0.08 (0.03,0.20)	<0.001	0.31 (0.11,0.93)	0.04	0.36 (0.12,1.06)	0.06
Trunk muscle index [kg/(kg/m^2^)]						
Continuous variable	0.27 (0.13,0.54)	<0.001	0.35 (0.16,0.78)	0.01	0.34 (0.16,0.75)	0.01
Categorical variable						
Quantile1	ref		ref		ref	
Quantile2	0.87 (0.68,1.13)	0.30	0.83 (0.63,1.09)	0.17	0.81 (0.61,1.07)	0.14
Quantile3	0.92 (0.71,1.20)	0.53	0.87 (0.66,1.16)	0.35	0.85 (0.65,1.13)	0.26
Quantile4	0.66 (0.52,0.83)	<0.001	0.69 (0.53,0.89)	0.01	0.68 (0.52,0.88)	0.004
*p* for trend		0.002		0.01		0.01

Model 1 was adjusted for none. Model 2 was adjusted for age, race, family income to poverty ratio, marital status, citizenship, and education attainment. Model 3 was adjusted for age, race, family income to poverty ratio, marital status, citizenship, education attainment, smoking status, drinking habits, hysterectomy, cancer, stroke, vigorous activities, moderate activities, coronary heart disease, congestive heart failure, previous pregnancies, diabetes, and hypertension.

**Figure 2:**
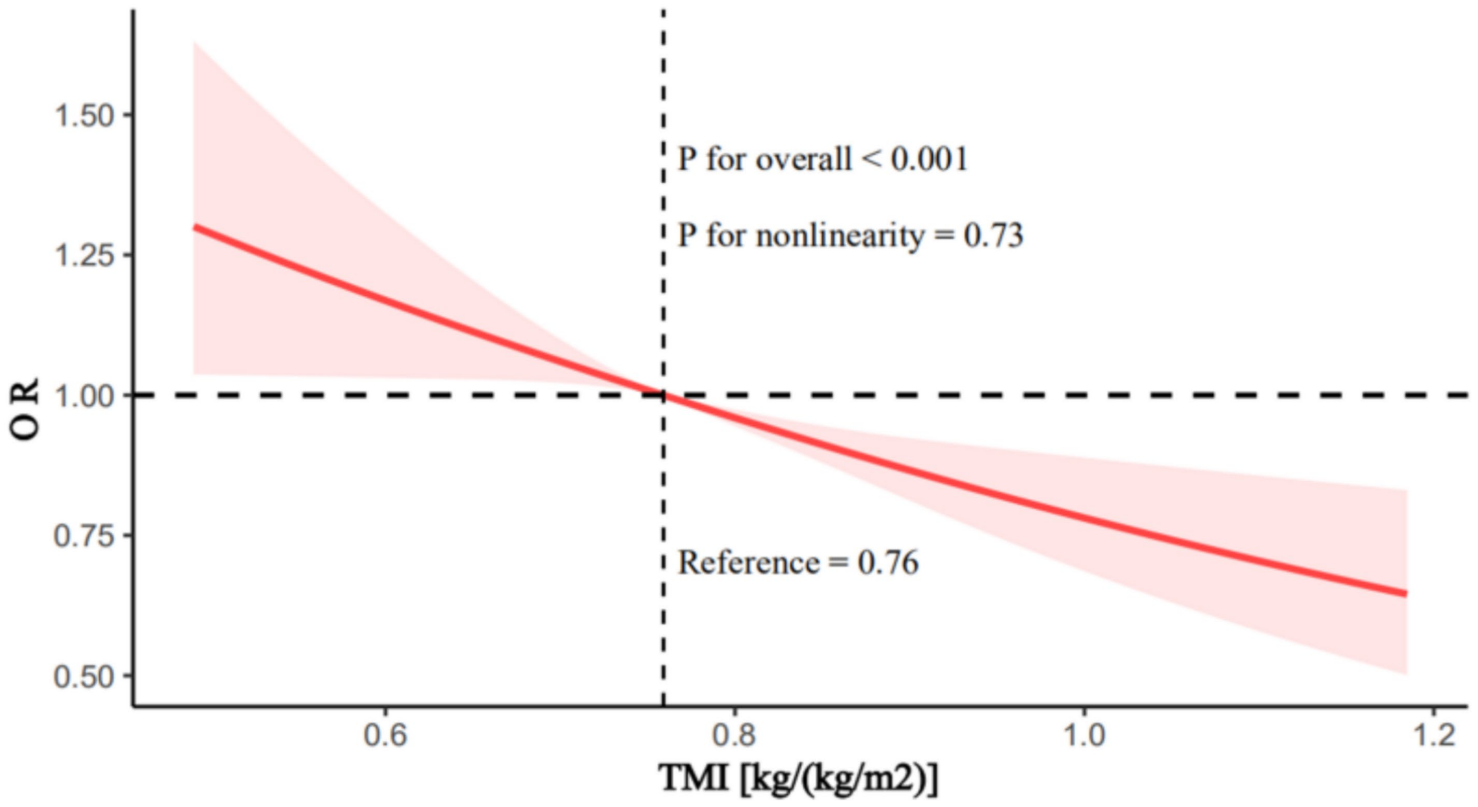
Restricted cubic splines analysis of the association between trunk muscle index and stress urinary incontinence. Adjusted for age, race, family income to poverty ratio, marital status, citizenship, education attainment, smoking status, drinking habits, hysterectomy, cancer, stroke, vigorous activities, moderate activities, coronary heart disease, congestive heart failure, previous pregnancies, diabetes, and hypertension. TMI, trunk muscle index.

The relationship between TMI and SUI remained closely linked across all three models. In Model 3, for each unit increase in TMI, the risk of SUI decreased by 66% (OR = 0.34; 95% CI: 0.16–0.75; *p* = 0.01). Relative to the first quartile of TMI, being in the fourth quartile reduced the risk of SUI by 31.3% (OR = 0.68; 95% CI: 0.52–0.88; *p* = 0.004). RCS analysis revealed a linear relationship between TMI and the risk of SUI, showing a decreasing trend in SUI risk as TMI increases (Figure 2; *p* for overall <0.001, *p* for nonlinearity = 0.73).

### 3.3 Subgroup analysis

Based on stratified analysis considering age, educational attainment, PIR, smoking status, vigorous activities, hypertension, diabetes, and hysterectomy, the interaction tests between TMI and SUI did not reveal any significant differences (*p* for interaction >0.05; Table 4). This indicates that the specified baseline traits had no effect on altering this association.

**Table 4:** Subgroup analyses of the association between trunk muscle index and stress urinary incontinence.

Subgroups	OR (95% CI)	*p* value	*p* for interaction
Age			0.24
20–39 years	1.26 (0.29,0.554)	0.76	
40–59 years	0.24 (0.07, 0.84)	0.03	
≥60 years	0.13 (0.01,1.31)	0.08	
Educational attainment			0.75
Below high school level	0.67 (0.04,12.33)	0.78	
College level and above	0.45 (0.15,1.30)	0.14	
High school level	0.16 (0.03, 0.89)	0.04	
PIR			0.40
<1.3	0.12 (0.02,0.82)	0.03	
1.3–3.4	0.15 (0.04, 0.52)	0.004	
≥3.5	0.67 (0.19,2.36)	0.53	
Smoke status			0.61
Former	1.14 (0.25,5.08)	0.86	
Never	0.20 (0.06,0.67)	0.01	
Current	0.41 (0.07, 2.54)	0.34	
Vigorous activities			0.64
No	0.27 (0.09,0.79)	0.02	
Yes	0.85 (0.15, 4.97)	0.86	
Hysterectomy			0.06
No	0.51 (0.22,1.16)	0.11	
Yes	0.12 (0.02, 0.64)	0.01	
Diabetes			0.94
No	0.36 (0.15,0.83)	0.02	
Yes	0.26 (0.00,15.63)	0.51	
Hypertension			0.37
No	0.28 (0.09,0.83)	0.02	
Yes	0.58 (0.12,2.77)	0.49	

Adjusted for age, race, family income to poverty ratio, marital status, citizenship, education attainment, smoking status, drinking habits, hysterectomy, cancer, stroke, vigorous activities, moderate activities, coronary heart disease, congestive heart failure, previous pregnancies, diabetes, and hypertension. When a stratified analysis of an independent variable is performed, the independent variable itself is not adjusted. OR, odd ratio; CI, confidence interval; PIR, family income to poverty ratio.

### 3.4 Stability analysis

To assess the impact of missing data on the results, this study conducted a multivariate logistic regression analysis of the relationship between TMI and SUI after including all missing data (Table 5). The results showed that the relationship between TMI and the risk of SUI remained significant (OR = 0.38; 95% CI: 0.24–0.62; *p* < 0.001).

**Table 5:** Association between trunk muscle index and stress urinary incontinence (including excluded missing data).

Exposure	Model 1		Model 2		Model 3	
	OR, 95%CI	*p*	OR, 95%CI	*p*	OR, 95%CI	*p*
Trunk muscle index [kg/(kg/m^2^)]						
Continuous variable	0.26 (0.17,0.39)	<0.001	0.33 (0.21,0.54)	<0.001	0.38 (0.24,0.62)	<0.01
Categorical variable						
Quantile1	ref		ref		ref	
Quantile2	1.07 (0.90,1.27)	0.45	1.03 (0.85,1.24)	0.76	1.02 (0.85, 1.23)	0.82
Quantile3	1.02 (0.86,1.20)	0.84	0.99 (0.82,1.18)	0.87	1.00 (0.83,1.19)	0.97
Quantile4	0.69 (0.60,0.80)	<0.001	0.74 (0.63,0.86)	<0.001	0.76 (0.66,0.89)	<0.01
*p* for trend		<0.001		<0.001		<0.01

Model 1 was adjusted for none. Model 2 was adjusted for age, race, family income to poverty ratio, marital status, citizenship, and education attainment. Model 3 was adjusted for age, race, family income to poverty ratio, marital status, citizenship, education attainment, smoking status, drinking habits, hysterectomy, cancer, stroke, vigorous activities, moderate activities, coronary heart disease, congestive heart failure, previous pregnancies, diabetes, and hypertension.

## 4 Discussion

This study obtained data from NHANES on appendicular skeletal muscle mass, trunk muscle mass, BMI, and SUI for 11,168 female participants aged 20 and above during the periods of 2001–2006 and 2011–2018. We found that in models 1 and 2, there was an inverse association between ASMI, TMI, and the risk of SUI; that is, as ASMI and TMI increased, the risk of SUI decreased. However, in model 3, which adjusted for all covariates, this association remained only for the effect of TMI on the risk of SUI. RCS analysis revealed a linear relationship between TMI and the risk of SUI, showing a decreasing trend in SUI risk as TMI increases. After including all missing data, a multivariable logistic regression analysis was conducted to examine the relationship between TMI and SUI. The results indicated that the relationship between TMI and the risk of SUI remained significant.

Sarcopenia or muscle mass and urinary incontinence may be influenced by certain similar factors, including the fact that urinary incontinence tends to increase with age, as sarcopenia is also an age-related condition (27). Moreover, smoking, diabetes, and hypertension are not only associated with the risk of urinary incontinence (15, 16, 29), but are also closely linked to sarcopenia (13, 14, 30). Therefore, this study selected these covariates for further subgroup analysis. Based on stratified analysis considering age, educational attainment, PIR, smoking status, vigorous activities, hypertension, diabetes, and hysterectomy, the effect of TMI on the risk of SUI is not influenced by these baseline characteristics.

Sarcopenia, often indicating a decline in muscle mass, strength, and function, was found to be closely related to human health (31). It not only reduced the quality of life but was also associated with an increased risk of frailty and physical disability, as well as chronic obstructive pulmonary disease, non-alcoholic fatty liver disease, and cardiovascular health (31–40). Patients with sarcopenia had higher rates of falls and hospitalizations (38). Muscle quantity or mass was a common measure for assessing sarcopenia (17). It could refer to the total skeletal muscle mass of the body or the muscle mass in different regions, such as the appendicular skeletal muscle mass (17), the muscle mass at the level of the third lumbar vertebra (L3) (41, 42), the mid-thigh muscle mass (43), and the psoas muscle mass (44). As previously noted, muscle mass was influenced by factors such as height, weight, or BMI, necessitating adjustment of the results according to these metrics (17). Adjusting for BMI might be more appropriate as it takes into account the influence of both height and weight. However, the indices related to muscle mass in different body regions were associated with various diseases or physical conditions. For instance, the index related to appendicular skeletal muscle mass was associated with cardiovascular health metrics (33), while the index related to muscle mass at the level of the third lumbar vertebra (L3) was associated with cancer (41, 42).

A study found that low muscle mass, defined as muscle mass adjusted by BMI for women <0.823 kg/BMI, elevated the likelihood of UI in women (OR = 1.971, 95% CI: 1.369–2.838, *p* < 0.001) (27). However, in this study, muscle mass was assessed using the skeletal muscle mass of the whole body, making it unclear whether the muscle mass of different body parts had varying effects on urinary incontinence. Additionally, the outcome measure of the study was UI, without distinguishing between types of urinary incontinence, such as SUI or urge urinary incontinence (UUI), which had different pathogenesis. SUI, for example, might have been more related to urethral-vaginal support damage and increased abdominal pressure (45). Therefore, our study attempted to investigate the relationship between ASMI and TMI with SUI. They, respectively, represented the muscle mass and BMI balance of the appendicular and trunk muscles (including pelvic muscles) in different body regions. As our study results showed, after adjusting for all covariates, there was no association between ASMI and the risk of SUI, while a high TMI reduced the risk of SUI. This suggested that the ratio of muscle mass and BMI in different body regions has varying effects on SUI.

It should also be noted that, due to the format of the NHANES data, this study used trunk muscle mass data, which included pelvic muscle mass as well as the skeletal muscle mass of the lumbar spine and thoracic spine. Therefore, the mechanism linking TMI and the risk of SUI might have been related not only to the ratio of pelvic muscle mass to BMI, but also to the ratio of lumbar and thoracic muscle mass to BMI. Atrophy or weakness of the pelvic muscles can cause changes in the pelvic structural angles, leading to SUI (27, 46). Moreover, pelvic muscle exercises effectively improved SUI (47, 48). Pelvic floor muscle training played a key role in the management of urinary incontinence in women (49, 50). Good pelvic muscle support enhanced sphincter control, effectively preventing and treating incontinence (51). A systematic review showed that pelvic muscle strengthening not only improved muscle strength but also reduced the incidence of urinary incontinence, contributing to the prevention of SUI (52). These studies suggested that pelvic muscles played an important role in the pathogenesis of SUI. Additionally, the muscles of the thoracic and lumbar spine played a crucial role in maintaining spinal stability. Although there had been no studies directly examining the impact of thoracic or lumbar spine muscle mass or quantity on SUI, preliminary research indicated that alterations in lumbar posture and overall spinal alignment could affect pelvic function and posture (53, 54). These changes might have indirectly influenced the incidence of SUI. Therefore, the muscle mass index of different body parts may be associated with various disease conditions. Limb muscles are mainly responsible for movement and strength, with relatively less impact on urinary control. In contrast, the trunk muscle mass, which includes pelvic floor muscle mass, has a more direct influence on SUI.

Based on the results of this study, strengthening trunk muscles (including pelvic muscles) may be more effective than interventions that focus solely on appendicular muscles. Incorporating training targeting the trunk and pelvic muscles into SUI intervention programs could reduce the incidence of SUI. It is recommended to conduct future clinical trials or longitudinal studies to further investigate whether trunk muscle strengthening interventions can reduce the risk of SUI in the population. Future research should focus on developing more accurate techniques to measure pelvic muscle quality in order to better understand its relationship with SUI risk. Additionally, in-depth studies on the interactions between pelvic muscle mass, function, and SUI risk are needed to identify potential biomarkers and intervention targets.

The substantial sample size of this study bolsters the representativeness of its findings. Moreover, the study utilized weighted multivariable logistic regression and RCS analyses to confirm the association between ASMI, TMI, and the risk of SUI. One of the main advantages of RCS was its ability to flexibly model nonlinear relationships, making it easier to visualize the relationship between TMI and the risk of SUI. RCS was designed to maintain a smooth and continuous fit, minimizing the risk of overfitting to noise in the data. Multivariable regression analysis allowed us to control for multiple potential confounding variables, thereby identifying the independent relationship between TMI/ASMI and the risk of SUI. If we had used univariable analysis, we might have overlooked the influence of other factors on the outcomes, leading to biased conclusions. In contrast, multivariable regression provided a more comprehensive perspective, enabling us to more accurately assess the contribution of ASMI or TMI to the risk of SUI. Stratified analysis was also applied to evaluate the potential effects of covariates on this association, thus strengthening the robustness of the research outcomes. However, the limitations of this study should not be overlooked. Firstly, as a cross-sectional study, it could not directly establish causality, necessitating longitudinal research for further exploration and validation. Secondly, the covariates included might not have been comprehensive. Specifically, NHANES did not provide information regarding muscle tissue quality, regular physical activity levels, whether SUI patients received treatment, as well as the type of treatment they received. Thirdly, some of the data on study variables were self-reported, which may introduce recall bias. Some participants might have underestimated or overestimated the frequency of their symptoms, which could have affected our assessment of SUI prevalence. Participants might also have found it difficult to accurately recall the timing of their symptoms. Lastly, NHANES did not provide separate data on pelvic muscle mass and muscle mass of other skeletal regions such as thoracic and lumbar spine, thereby limiting the study of whether the muscle mass in these different areas has a differential impact on SUI. Additionally, in this study, some participants were excluded from the analysis due to missing data. A comparison of the characteristics between the excluded participants and those included in the study revealed differences in several aspects between the two groups. These differences suggested that the included sample might not have fully represented the entire target population. Future research should have aimed to minimize the occurrence of missing data to ensure the diversity of the study sample and the generalizability of the results. In this study, a formal statistical power analysis was not conducted. Although the sample size was relatively large and preliminary analyses showed some significant associations, the lack of a power analysis made it impossible to clearly determine whether the sample size was sufficient to support the conclusions of this study.

## 5 Conclusion

As far as we are aware, this study was the first to examine the connection between ASMI, TMI, and the likelihood of developing SUI. The results of this study indicated that after controlling for other covariates, no correlation was found between ASMI and the likelihood of SUI, while TMI might have served as a protective factor against SUI. This suggested that the impact of muscle mass from different body regions relative to BMI on SUI varied. Therefore, in clinical practice, the ratio of trunk muscle mass to BMI could be used to assess the risk of SUI without the need to calculate the skeletal muscle mass of the entire body, which could reduce the workload for clinicians. Additionally, it was necessary to further investigate the muscle mass of the pelvis and regions such as the lumbar or thoracic spine in future studies, which could have provided more precise guidance for clinical practitioners.

## Data Availability

Publicly available datasets were analyzed in this study. This data can be found here: https://www.cdc.gov/nchs/nhanes/index.htm.

## Footnotes

^1^https://wwwn.cdc.gov/nchs/nhanes/default.aspx

^2^https://wwwn.cdc.gov/nchs/nhanes/analyticguidelines.aspx
